# Glycerol: An unexpected major metabolite of energy metabolism by the human malaria parasite

**DOI:** 10.1186/1475-2875-8-38

**Published:** 2009-03-06

**Authors:** Lu-Yun Lian, Mohammed Al-Helal, Abd Majid Roslaini, Nicholas Fisher, Patrick G Bray, Stephen A Ward, Giancarlo A Biagini

**Affiliations:** 1 School of Biological Sciences, Biosciences Building, University of Liverpool, P.O. Box 147, Liverpool, L69 7ZB, UK; 2 Liverpool School of Tropical Medicine, Liverpool, L35 QA, UK

## Abstract

**Background:**

Malaria is a global health emergency, and yet our understanding of the energy metabolism of the principle causative agent of this devastating disease, *Plasmodium falciparum*, remains rather basic. Glucose was shown to be an essential nutritional requirement nearly 100 years ago and since this original observation, much of the current knowledge of *Plasmodium *energy metabolism is based on early biochemical work, performed using basic analytical techniques (e.g. paper chromatography), carried out almost exclusively on avian and rodent malaria. Data derived from malaria parasite genome and transcriptome studies suggest that the energy metabolism of the parasite may be more complex than hitherto anticipated. This study was undertaken in order to further characterize the fate of glucose catabolism in the human malaria parasite, *P. falciparum*.

**Methods:**

Products of glucose catabolism were determined by incubating erythrocyte-freed parasites with D-[1-^13^C] glucose under controlled conditions and metabolites were identified using ^13^C-NMR spectroscopy.

**Results:**

Following a 2 h incubation of freed-*P. falciparum *parasites with 25 mM D-[1-^13^C] glucose (*n *= 4), the major metabolites identified included; [3-^13^C] lactate, [1,3-^13^C] glycerol, [3-^13^C] pyruvate, [3-^13^C] alanine and [3-^13^C] glycerol-3-phosphate. Control experiments performed with uninfected erythrocytes incubated under identical conditions did not show any metabolism of D-[1-^13^C] glucose to glycerol or glycerol-3-phosphate.

**Discussion:**

The identification of glycerol as a major glucose metabolite confirms the view that energy metabolism in this parasite is more complex than previously proposed. It is hypothesized here that glycerol production by the malaria parasite is the result of a metabolic adaptation to growth in O_2_-limited (and CO_2 _elevated) conditions by the operation of a glycerol-3-phosphate shuttle for the re-oxidation of assimilatory NADH. Similar metabolic adaptations have been reported previously for other microaerobic/anaerobic organisms, such as yeast, rumen protozoa and human parasitic protozoa.

**Conclusion:**

These data highlight the need to re-evaluate the carbon and redox balance of this important human pathogen, ultimately leading to a better understanding of how the parasite is able to adapt to the variable environments encountered during parasite development and disease progression.

## Background

Despite the clinical and economic significance of the human malaria parasite, *Plasmodium falciparum*, the energy metabolism of this organism is still poorly understood. Glucose was shown to be an essential nutritional requirement nearly 100 years ago [1] and experiments performed using [^14^C]-glucose revealed that lactate is the major detectable end product of glucose catabolism together with a variety of other organic acids (e.g. pyruvate, acetate, succinate, aspartate, glutamate, alanine and CO_2_) depending on the species of malaria [2]. It is perhaps surprising to note that much of this early biochemical work was performed almost exclusively on avian and rodent malaria and very little information exists regarding human malaria parasites.

It is clear from genome sequence information, that the energy metabolism of *P. falciparum *is more complex than hitherto anticipated and an almost full compliment of tricarboxylic acid (TCA) cycle and electron transport chain (ETC) genes have so far been identified [3-5]. Subsequent transcriptome data indicates an up-regulation of expression of many of these genes during the transition from asexual to sexual stages [6-8] and more recently there is evidence of different expression profiles of *in vivo *intraerythrocytic parasites compared to laboratory cultured parasites [9].

However, without biochemical data the role of each of these components and their interdependence remains hypothetical. This study has, therefore, set out to perform a qualitative analysis of glucose catabolism using ^13^C-NMR. The advantage of this technique is that it allows the identification of metabolites without the *a priory *need of information regarding the products. This study reports that glycerol is an unexpected product of glucose catabolism in the human malaria parasite *P. falciparum*, and the role played by this pathway in maintaining the redox balance of the cell is discussed.

## Methods

### Parasite culture and free parasite preparation

*Plasmodium falciparum *strain 3D7 was maintained in continuous culture in a 2% suspension of O+ erythrocytes in RPMI 1640 medium (R8758, glutamine, and NaHCO3) supplemented with 10% pooled human serum, 25 mM HEPES (pH 7.4), and 20 μM gentamicin sulphate[10].

Preparation of free parasites, from an aliquot of infected erythrocytes, was performed by centrifugation (3,000 × g, 5 min) and resuspension of the pellet in 5 vol of 0.15% (wt/vol) saponin in phosphate-buffered saline (PBS) for 1 min, followed by three washes by centrifugation and resuspension in Ringers (106 mM NaCl, 24 mM NaHCO_3_, 5.4 mM KCl, 1.2 mM CaCl_2_, 1 mM Na_2_HPO_4_, 0.8 mM MgCl_2_, 5.5 mM D-glucose, pH 7.4) and two washes in glucose-free Ringers. Samples were checked microscopically to ensure lysis of all erythrocytes. It is worth noting that there is a large body of evidence to show that *P. falciparum *parasites isolated using the saponin lysis technique remain viable for at least 2 h as demonstrated by the maintenance of the parasite plasma membrane potential [11], maintenance of the ATP/ADP couple [12] and the operation of a number of H+- and Na+-dependent transporters, which can only operate when the integrity of the plasma membrane electrochemical gradient is maintained e.g. [13-15].

### NMR spectroscopy measurements

Products of glucose fermentation were identified by incubating erythrocyte freed-parasites at 37°C under 3% O_2_/4% CO_2 _in N_2 _for 2 h, in Ringers (pH 7.4) containing 25 mM D-[1-^13^C] glucose. Following incubation, cell suspensions were disrupted by probe sonication, centrifuged (13 000 *g *× 2 min) and the supernatants collected for analyses. Proton-decoupled ^13^C-NMR spectra were recorded at 600 MHz on a Bruker Avance spectrometer equipped with a triple resonance cryoprobe. Free induction decay was measured for a total of 26,000 data points covering a spectral width of 197 ppm with pulses of 10.3 ms (90°) at 2 s intervals. ^2^H_2_O was used as the internal lock. Chemical shifts, in parts per million, were measured with respect to the bC-1 resonance in the added D-glucose (97.0 ppm) [16]. Data presented are representative of 4 independent experiments. Spectra were also recorded for control experiments performed under identical conditions with uninfected human erythrocytes (5% suspension).

## Results and Discussion

**Figure 1:**
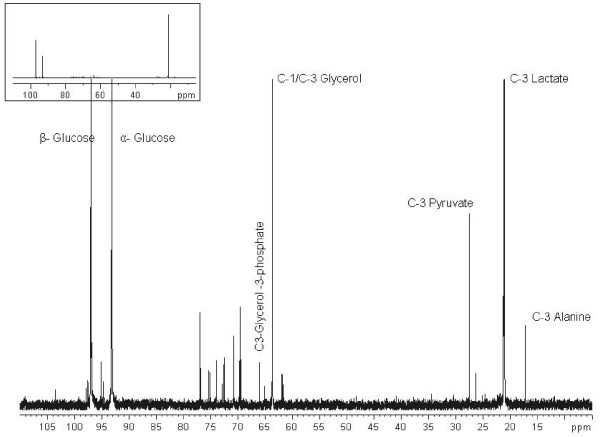
**Proton-decoupled ^13^C-NMR spectrum of erythrocyte-freed *P. falciparum *following incubation with D-[1-^13^C] glucose (25 mM)**. Chemical shifts in parts per million were as follows: C-1 α-glucose, 93.2; C-1 β-glucose, 97.0; C-3 lactate, 21.2; C-1,3 glycerol, 63.6; C-3 pyruvate, 27.6; C-3 alanine, 17.3 and C-3 glycerol-3-phosphate, 65.9. Spectrum representative of a typical experiment (*n *= 4).

It has long been the held view that lactate is the sole end-product of glucose fermentation by *P. falciparum *[2]. By following the catabolism of D-[1-^13^C] glucose by ^13^C-NMR, this study has identified that in addition to lactate, glycerol (and glycerol phosphate), pyruvate and alanine are significant end products of glucose fermentation. A typical ^13^C-NMR spectrum of erythrocyte-freed trophozoite stage *P. falciparum *following 2 h incubation with 25 mM D-[1-^13^C] glucose is shown in Fig. 1. Metabolites were identified according to previously reported shifts (ppm)[16], these included [3-^13^C] lactate (21.2 ppm), [1,3-^13^C] glycerol (63.6 ppm), [3-^13^C] pyruvate (27.6 ppm), [3-^13^C] alanine (17.3 ppm) and [3-^13^C] glycerol-3-phosphate (65.9 ppm) and also by spiking test samples with known reference compounds (performed for each experiment, *n *= 4). Control experiments performed with uninfected erythrocytes incubated under identical conditions did not show any metabolism of D-[1-^13^C] glucose to glycerol or glycerol-3-phosphate.

The identification of additional glucose catabolism end-products raises several questions regarding our understanding of the carbon and redox balance of the malaria parasite. Glycerol has previously been postulated to be taken up by the malaria parasite from the host serum for the synthesis of lipids and membranes and recently the crystal structure of the aquaglycerolporin PfAQP, involved in the transport of glycerol, was solved [17]. However, data from this study suggest that glycerol can be generated by the malaria parasite during asexual growth. The formation of glycerol is unusual amongst eukaryotes (note glycerol is not produced by uninfected human erythrocytes [18], nor was it detected in control experiments), although it is a feature of anaerobic glucose catabolism in yeast [19], protozoan parasites such as trichomonas [20], leishmania [21] and trypanosomes [22], as well as rumen ciliates such as Dasytricha [23], Eudiplodinium [24], and Polyplastron [25]. Interestingly, in all of these studies ^13^C-NMR spectroscopy was the method adopted for the identification of glycerol. As glycerol is a poor chromophore and ionizes poorly in an ESI (electro-spray ionisation) source, complementary techniques such as HPLC or GC-MS/MS and LC-MS/MS are either insensitive or non-selective. These problems can only be overcome with extensive derivatization of glycerol (e.g[26]). The relative analytical difficulties associated with detecting glycerol may explain why this metabolite has previously gone undetected in *Plasmodium*.

During anaerobic glycolysis, it is believed that glycerol is generated for the purposes of restoring the cell's redox balance. This occurs via an indirect mechanism known as the glycerol-3-phosphate shuttle that results in the oxidation of NADH and the transfer of electrons to the ETC. The shuttle consists of two components, cytosolic glycerol-3-phosphate dehydrogenase and mitochondrial glycerol-3-phosphate:ubiquinone oxidoreductase, both these components are present in *P. falciparum *[3]. At first sight, the need for such a shuttle to operate in *P. falciparum *may be questioned, as the production of lactate from glucose is redox neutral. However, as demonstrated from the study of yeast, growth (e.g. biomass production) is essentially a reductive process resulting in a net consumption of NADPH and a net production of NADH [19]. As a result cells have developed a number of mechanisms to re-oxidize NADH in various compartments. It would appear from data presented here that the malaria parasite possess a glycerol-3-phosphate shuttle to reoxidize cytosolic NADH whereas mitochondrial NADH may be re-oxidized using an alternative mechanism such as the type II NADH:ubiquinone oxidoreductase (alternative complex I) [27,28].

At this stage it is uncertain how the malaria parasite generates glycerol. In yeast, glycerol is produced from the hydrolysis of the phosphate group of glycerol-3-phosphate via glycerol-3-phosphatase [29], however this enzyme appears to be missing from the genome of the human malaria parasite [3]. In trypanosomes, glycerol is generated from glycerol kinase operating in reverse with the concomitant formation of ATP [30-32]. In trypanosomes, this reaction occurs inside glycosomes where it is believed that the compartmentalisation of substrates at un-physiological concentrations (relative to the cytosol) allows this otherwise thermodynamically unfavourable reaction to take place [30,31]. Whether or not the *P. falciparum *glycerol kinase is similarly able to operate in reverse remains to be determined, however preliminary data would suggest that anaerobic incubation of *P. falciparum *parasites results in an increase in the intracellular levels of glycerol-3-phosphate (Biagini, unpublished).

## Conclusion

This study shows that *P. falciparum *asexual parasites are able to generate glycerol from glucose. The production of glycerol is believed to be a metabolic adaptation to growth in O_2_-limited environments. These data support previous hypotheses that mitochondrial dehydrogenases such as PfNDH2 and G3PDH are important to the parasite for the purposes of redox balance under conditions of low O_2 _[28,33,34].

## Competing interests

The authors declare that they have no competing interests.

## Authors' contributions

L-YL carried out the NMR spectroscopy. MAH, MR, NF and GAB carried out the parasite culture, ^13^C-glucose incubations and sample preparations. PGB and SAW participated in the design of the study and drafting of the manuscript. GAB conceived the study, and participated in its design and coordination and prepared the manuscript. All authors read and approved the final manuscript.
